# Postexercise muscle glycogen synthesis with glucose, galactose, and combined galactose-glucose ingestion

**DOI:** 10.1152/ajpendo.00127.2022

**Published:** 2023-10-18

**Authors:** Tim Podlogar, Brandon J. Shad, Alex P. Seabright, Oliver J. Odell, Samuel O. Lord, Rita Civil, Rafael B. Salgueiro, Emma L. Shepherd, Patricia F. Lalor, Yasir S. Elhassan, Yu-Chiang Lai, David S. Rowlands, Gareth A. Wallis

**Affiliations:** ^1^School of Sport, Exercise and Rehabilitation Sciences, College of Life and Environmental Sciences, University of Birmingham, Birmingham, United Kingdom; ^2^Department of Physiology and Biophysics, University of Sao Paulo, Sao Paulo, Brazil; ^3^Centre for Liver and Gastroenterology Research and National Institute for Health Research Birmingham Biomedical Research Centre, Institute of Immunology and Immunotherapy, University of Birmingham, Birmingham, United Kingdom; ^4^Institute of Metabolism and Systems Research, University of Birmingham, Birmingham, United Kingdom; ^5^Centre for Endocrinology, Diabetes and Metabolism, Birmingham Health Partners, Birmingham, United Kingdom; ^6^School of Sport, Exercise and Nutrition, Massey University, Auckland, New Zealand

**Keywords:** exercise, nutrition, recovery, sugars

## Abstract

Ingested galactose can enhance postexercise liver glycogen repletion when combined with glucose but effects on muscle glycogen synthesis are unknown. In this double-blind randomized study participants [7 men and 2 women; V̇o_2max_: 51.1 (8.7) mL·kg^−1^·min^−1^] completed three trials of exhaustive cycling exercise followed by a 4-h recovery period, during which carbohydrates were ingested at the rate of 1.2 g·kg^−1^·h^−1^ comprising glucose (GLU), galactose (GAL) or galactose + glucose (GAL + GLU; 1:2 ratio). The increase in vastus lateralis skeletal-muscle glycogen concentration during recovery was higher with GLU relative to GAL + GLU [contrast: +50 mmol·(kg DM)^−1^; 95%CL 10, 89; *P* = 0.021] and GAL [+46 mmol·(kg DM)^−1^; 95%CL 8, 84; *P* = 0.024] with no difference between GAL + GLU and GAL [−3 mmol·(kg DM)^−1^; 95%CL −44, 37; *P* = 0.843]. Plasma glucose concentration in GLU was not significantly different vs. GAL + GLU (+ 0.41 mmol·L^−1^; 95%CL 0.13, 0.94) but was significantly lower than GAL (−0.75 mmol·L^−1^; 95%CL −1.34, −0.17) and also lower in GAL vs. GAL + GLU (−1.16 mmol·^−1^; 95%CL −1.80, −0.53). Plasma insulin was higher in GLU + GAL and GLU compared with GAL but not different between GLU + GAL and GLU. Plasma galactose concentration was higher in GAL compared with GLU (3.35 mmol·L^−1^; 95%CL 3.07, 3.63) and GAL + GLU (3.22 mmol·L^−1^; 95%CL 3.54, 2.90) with no difference between GLU + GAL (0.13 mmol·L^−1^; 95%CL −0.11, 0.37) and GLU. Compared with galactose or a galactose + glucose blend, glucose feeding was more effective in postexercise muscle glycogen synthesis. Comparable muscle glycogen synthesis was observed with galactose-glucose coingestion and exclusive galactose-only ingestion.

**NEW & NOTEWORTHY** Postexercise galactose-glucose coingestion or exclusive galactose-only ingestion resulted in a lower rate of skeletal-muscle glycogen replenishment compared with exclusive glucose-only ingestion. Comparable muscle glycogen synthesis was observed with galactose-glucose coingestion and exclusive galactose-only ingestion.

## Graphical abstract

**Figure:**
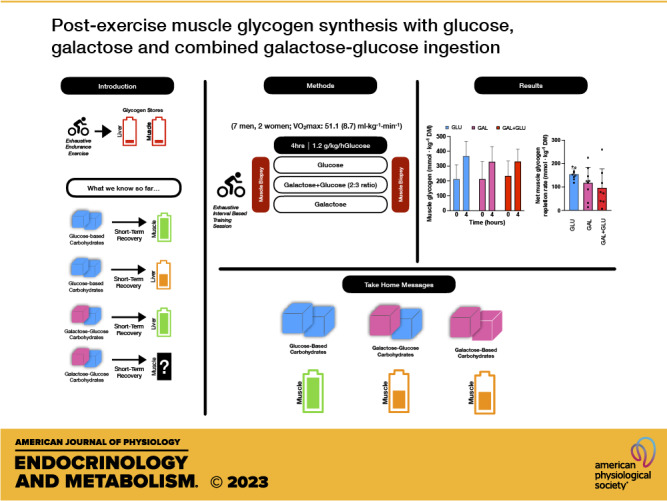

## INTRODUCTION

The importance of skeletal-muscle glycogen for endurance exercise is well established (1–3). Exhaustive endurance exercise results in substantial reductions in skeletal-muscle glycogen content (4). Following exhaustive endurance exercise, at least 24 h of high dietary-carbohydrate ingestion is required to attain complete repletion of skeletal-muscle glycogen content (5). Accordingly, nutritional strategy is particularly important when the time between successive exercise bouts is limited (e.g., 2–8 h). The impact of the dose and the timing of carbohydrate intake on skeletal-muscle glycogen synthesis during the postexercise recovery time window is well investigated, with saturation of repletion rates achieved when carbohydrates are ingested at the rate of ≥1.2 g per kilogram body mass per hour (g·kg^−1^·h^−1^) (6). More recently, in acknowledging the importance of the liver for the maintenance of glycemia during exercise, research has been directed toward developing nutritional strategies that optimize both skeletal muscle and hepatic glycogen synthesis (7, 8). These efforts have focused attention on how the type of carbohydrate ingested, more specifically the constituent monosaccharides, can influence short-term glycogen synthesis in muscle and liver.

The evidence suggests that following prolonged endurance exercise the ingestion of combined fructose-glucose blends as compared with exclusively glucose-based carbohydrates results in similar skeletal-muscle glycogen synthesis outcomes, but higher liver glycogen synthesis within the short-term (i.e., ≤5 h) recovery period (9–13). Ingesting exclusively fructose provides for substantially lower skeletal-muscle glycogen resynthesis, compared with glucose or sucrose (14). Indeed, while glucose is readily metabolized to provide energy substrate within skeletal-muscle tissue, the majority, but not all the fructose carbon (15–17) first needs to be metabolized in the liver to glucose before being available to other tissues creating a time delay for delivery to the muscle and greater carbon sequestration to metabolic substrates within the liver (18). In addition, combined galactose-glucose coingestion has been shown to provide a substrate for the repletion of liver glycogen stores in a quantitatively similar amount to fructose and glucose coingestion (13). However, the effects of galactose plus glucose or exclusive galactose ingestion on short-term postexercise skeletal-muscle glycogen have, to the authors knowledge, not been studied.

Galactose is mostly consumed within the human diet as lactose is present in milk and to a lesser degree galactose is found in various fruits, vegetables, and legumes (19). Accordingly, galactose most often presents in the intestine alongside glucose. At the intestinal epithelia, galactose is absorbed using the same transporters as glucose [i.e., sodium glucose-linked transporter 1, SLGT1; and glucose transporter 2, GLUT2 (20)]. Thereafter, metabolism in the liver via the Leloir pathway to UDP-glucose is considered the major route for galactose disposal (21). Given that fructose and galactose both preferentially undergo hepatic metabolism, we hypothesized that relative to glucose, exclusive galactose ingestion would elicit an inferior short-term postexercise skeletal-muscle glycogen synthesis rate (14). Furthermore, in a similar fashion to that observed with fructose-glucose blends, combined galactose-glucose ingestion would result in comparable short-term postexercise skeletal-muscle glycogen synthesis, relative to exclusive glucose provision (9, 14).

Therefore, the primary objective of the present study was to compare the effects of glucose, galactose, and combined galactose-glucose ingestion following exhaustive endurance exercise on skeletal-muscle glycogen synthesis during the short-term recovery period (i.e., 4 h). The study also investigated how the nutritional interventions affected selected plasma metabolic and skeletal muscle molecular signaling responses and, finally, in an exploratory analysis, the expression of Leloir pathway enzymes was probed in human skeletal muscle.

## METHODS

### Participant Characteristics

Nine participants (7 men and 2 women) provided written informed consent and completed the study that was approved by the West Midlands—Black Country Research Ethics Committee, United Kingdom (REC/NRES Study Number: 19/WM/0150) and registered at clincialtrials.gov (NCT03903861). Participants had to meet basic inclusion criteria, i.e., be healthy, aged 18–45 yr, participate in regular endurance exercise, and have a V̇o_2peak_ >45 and >40 mL·kg^−1^·min^−1^ for men and women, respectively. Their characteristics are displayed in Table 1.

**Table 1.** Participant characteristics

Age, yr	26 (8)
Body mass, kg	70.6 (8.6)
Height, cm	175 (10)
V̇o_2peak_, L·min^−1^	3.6 (0.8)
V̇o_2peak_, mL·kg^−1^·min^−1^	51.1 (8.7)
Wmax, W	330 (80)
Wmax, W·kg^−1^	4.7 (1.0)

Data are means (SD). V̇o_2peak_, peak oxygen uptake; Wmax, maximal cycle ergometer power output.

### Experimental Design

After preliminary testing, each participant completed a familiarization and three experimental trials. Each experimental trial commenced with an exhaustive, glycogen-reducing exercise bout and was followed by a 4-h long recovery period during which carbohydrate drinks were provided. Trials differed in the type of carbohydrates provided to participants. Participants received glucose (GLU), galactose (GAL), or galactose-glucose (GAL-GLU). The study was double-blinded, and the order of the trials was randomized using the free online research randomizer software (www.randomizer.org). Muscle glycogen concentrations were determined from skeletal muscle biopsies obtained from the vastus lateralis muscle before a glycogen-reducing exercise bout during the familiarization session (i.e., estimate of typical resting glycogen content) and then in each experimental trial immediately after the exhaustive exercise bout and following the 4-h recovery period. Venous blood samples were collected during the recovery period and subsequently analyzed for selected metabolites and insulin.

In an exploratory analysis, the gene and protein expressions of key enzymes of the Leloir pathway were probed in skeletal muscle biopsy samples obtained in the present study. HepG2 cell or human liver tissue lysates were used as positive controls. Human liver tissue was collected at the Liver and Hepatobiliary Unit, Queen Elizabeth Hospital, Birmingham, with prior written informed patient consent and local research ethics committee approval (REC/NRES Study Numbers 19/WA/0139; 06/Q702/61). Normal nondiseased liver was obtained from donor tissue not suitable for transplantation.

### Preliminary Testing and Familiarization Trial

Participants performed an incremental exercise test to task failure to determine V̇o_2peak_ and Wmax on a cycle ergometer (Excalibur Sport; Lode, Groningen, the Netherlands), as previously described (22). In brief, the test started at a power output of 100 W and the workload increased by 30 W every 2 min. During the test, gas exchange measurements were made using an automated online gas analysis system (Vyntus, Vyaire Medical, Ottawa, IL). The highest 30-s average of O_2_ uptake was considered to represent V̇o_2peak_. Wmax was calculated as the power output from the last completed stage plus the fraction of the time spent in the next stage multiplied by 30 W.

Participants meeting the study’s minimal V̇o_2peak_ criteria were then scheduled for the familiarization trial. This trial differed from the experimental trials in that only one muscle biopsy was taken, and this was before the glycogen-reducing exercise session to obtain an indication of typical resting glycogen concentration.

### Experimental Trials

Participants entered the laboratory at ∼0700 after not eating from 2200 the day before. They were asked to replicate the diet and activity patterns of the day preceding each experimental trial. The trials started with a glycogen-reducing exercise as described previously (9, 23) formerly shown to effectively reduce muscle glycogen concentration (9). In brief, after a 5-min warm-up at 50% Wmax participants cycled at alternating workloads of 90% and 50% Wmax, respectively, each lasting 2 min. Once the 90% workload was deemed too demanding for participants to be able to cycle at a cadence of more than 60 revolutions per minute (rev·min^−1^) despite strong verbal encouragement, the 90% intensity was first reduced to 80% and then to 70%. When blocks at 70% Wmax could not be completed at the cadence > 60 rev·min^−1^, the exercise session was terminated.

Participants then dismounted the ergometer and lay supine on a bed and the first muscle biopsy was obtained using the suction modified Bergström needle biopsy technique (24) from the vastus lateralis muscle through a skin incision under a local anesthesia (1% lidocaine). Following the procedure, an indwelling catheter was placed in an antecubital arm vein and the first venous blood sample was obtained.

Immediately upon obtainment of the blood sample, participants ingested an initial bolus (460 mL) of a carbohydrate-containing solution. During recovery, participants passively rested for 4 h, during which sedentary activities such as reading, and use of laptops were permitted. 220 mL doses of the test beverage were provided every 30 min during recovery such that the total fluid intake during the recovery period was 2.0 L. Drinks provided during the recovery period supplied carbohydrates at an average rate of 1.2 g·kg^−1^·h^−1^ with the composition of the drink either GLU (BulkPowders, Essex, UK), GAL (Galaxtra, Solace Nutrition, Connecticut) or GAL-GLU in a 1:2 ratio.

A venous blood sample was obtained every hour during the recovery with the cannula flushed with saline every 30 min to maintain patency. 4 h after the ingestion of the first carbohydrate-containing drinks a second muscle biopsy was obtained from the contralateral leg. Muscle biopsies in subsequent visits were taken from separate incisions in an alternating pattern between legs.

### Muscle Glycogen Analysis

On collection, muscle biopsy samples were immediately dissected free of visible fat and connective tissue and frozen in liquid nitrogen, before being stored at −70°C and subsequently powdered and freeze-dried. 2–5 mg of dry muscle (DM) tissue was hydrolyzed by adding 500 µL of 1 mmol·L^−1^ HCl and subsequently incubated for 2 h in an oven at 95°C. After cooling to room temperature, samples were neutralized with 2 M NaOH. This was followed by centrifugation (1,800 *g* at 4°C for 10 min) and the supernatant was analyzed in duplicate for glucose using an automated photometric-based clinical chemistry analyzer (described below). Where tissue size permitted (∼50%), muscle glycogen concentration was determined in duplicate. The intraassay coefficient of variation for muscle glycogen determination was <10%.

### Cell Lines and Culture

HepG2 cells were purchased from Tissue Culture Collection (ATCC, #CRL-10741). Cells were seeded on 100 mm dishes and cultured in Dulbecco’s modified Eagle medium (DMEM) containing GlutaMAX, 25 mM glucose, 1 mM sodium pyruvate, supplemented with 10% (vol/vol) fetal bovine serum, and 1% (vol/vol) Penicillin-Streptomycin (10,000 Units/mL-μg/mL). Medium (10 mL) was changed every other day. Cultures were maintained in a humidified incubator at 37°C with an atmosphere of 5% CO_2_ and 95% air.

### Cell Lysis

Cells were washed twice in ice-cold Dulbecco’s phosphate-buffered saline (DPBS) without calcium and magnesium. Cells were lysed in ice-cold sucrose lysis buffer (500 µL) containing: 250 mM sucrose, 50 mM Tris-base (pH 7.5), 50 mM sodium fluoride, 10 mM sodium β-glycerolphosphate, 5 mM sodium pyrophosphate, 1 mM EDTA, 1 mM EGTA, 1 mM benzamidine, 1 mM of sodium orthovanadate, 1 × complete Mini EDTA-free protease inhibitor cocktail, 1% (vol/vol) Triton X-100, and 100 mM 2-chloroacetamide. Cell lysates were centrifuged for 15 min at 8,000 *g* (4°C) and supernatant was stored at −70°C until further analysis.

### Tissue Homogenization

Using a TissueLyser II, freeze-dried, powdered muscle samples were disrupted in ice-cold sucrose lysis buffer by 5 mm stainless steel beads during 3 × 2 min cycles at 20 Hz. A 10-fold volume excess of ice-cold sucrose lysis buffer was used to lyse each sample. Resulting mixed muscle lysates were centrifuged for 10 min at 8,000 *g* (4°C) and the supernatant was stored at −70°C before further analysis. Snap frozen liver tissue (∼30 mg tissue) was lysed in 400 uL RIPA buffer plus PhosSTOP and cOmplete ULTRA (1 tablet in 10 mL RIPA buffer). Lysates were disrupted in Fisherbrand Pre-Filled Hard Tissue Ceramic bead mill tubes (2 mL capacity, 2.8 mm particle size) using Omni Bead Ruptor 12 for 3 cycles of 4 m/s speed, 20 s. Lysate suspension was centrifuged at 10,000 *g* for 5 min to pellet debris and lysate supernatant was stored at −70°C before further analysis.

### Protein Assay

Analysis of total protein was made using the Bradford protein assay. Briefly, 10 µL of cell or tissue lysate was diluted in 90 µL or 190 µL ddH2O, respectively. Diluted samples were loaded in duplicate into a 96-well microplate containing 300 µL Bradford protein reagent. Absorbance was measured at 595 nm using the FLUOstar OMEGA microplate reader. Protein in each sample was quantified from a standard curve using BSA standards ranging from 1 to 100 µg.

### Sample Preparation

Cell and tissue lysates were prepared in 1× NuPAGE LDS sample buffer containing 2-mercaptoethanol (final concentration 1.5%) and left to denature overnight at room temperature.

### Western Blot

Western ready samples were loaded into 4%–12% Bis/Tris precast gels (Invitrogen) or 10% homemade Bis/Tris gels before sodium dodecyl sulfate-polyacrylamide gel electrophoresis (SDS-PAGE). Gels were run in 1× MOPS buffer for approximately 80 min at 150 V. Proteins were transferred onto 0.45 µm PVDF membranes for 1 h at 100 V. Membranes were blocked in 3% of BSA diluted in Tris-buffered saline Tween-20 (TBS-T): 137 mM of sodium chloride, 20 mM of Tris-base (pH 7.5), 0.1% of Tween-20 for 1 h and incubated overnight at 4°C with the appropriate primary antibody. Primary antibodies were diluted in 3% of BSA made up in TBS-T (see Table 2). Membranes were washed in TBS-T three times before incubation in a horseradish peroxidase-conjugated secondary antibody (see Table 2) at room temperature for 1 h. Membranes were washed a further three times in TBS-T before antibody detection using enhanced chemiluminescence horseradish peroxidase substrate detection kit. Imaging was undertaken using a G:BOX Chemi-XR5. Quantification was performed using ImageJ.

**Table 2.** Antibodies used in Western blot analysis

Antibody	Source	Catalog Number	Concentration
Akt			
Protein kinase B	Cell Signaling Technology	4691	1:5,000
pSer473 Akt			
Protein kinase B phosphorylation site Ser473	Cell Signaling Technology	4060	1:5,000
pThr308 Akt			
Protein kinase B phosphorylation site Thr308	Cell Signaling Technology	2965	1:5,000
GS			
Glycogen Synthase	Cell Signaling Technology	3886	1:10,000
pSer641 GS			
Glycogen synthase Phosphorylation site Ser641	Cell Signaling Technology	3891	1:10,000
pSer21/9 α/β GSK3			
Phospho-glycogen-synthase-3-3alpha/beta (Ser21/9)	Cell Signaling Technology	9331	1:1,000
GAPDH			
Glyceraldehyde-3-phosphate dehydrogenase	Cell Signaling Technology	5174	1:20,000
Rabbit IgG, HRP-linked			
Anti-rabbit immunoglobin G, Horseradish Peroxidase-linked Antibody	Cell Signaling Technology	7074	1:10,000
GALK1			
Galactokinase 1	Invitrogen	PA5-90338	1:1,000
GALT			
Galactose-1-phosphate uridylyltransferase	Abcam	Ab178406	1:1,000
GALE			
UDP-galactose-4-epimerase	Abcam	Ab155997	1:1,000

### Gene Expression Analysis

RNA was extracted and purified from a sample of HepG2 cells using the ReliaPrep RNA Cell Miniprep System (Promega, Madison, WI), following the manufacturer’s instructions. RNA was extracted from two samples of ∼20 mg of frozen powdered human muscle tissue by homogenization in 1 mL of TRI reagent (Sigma-Aldrich, St Louis, MO) using an IKA T10 basic ULTRATURRAX homogenizer (IKA, Oxford, UK). To achieve phase separation, 200 μL of chloroform was added followed by vigorous shaking for 15 s, left standing for 15 min at ambient temperature, and subsequently centrifuged at 12,000 *g* for 15 min at 4°C. The RNA phase was then removed and mixed with an equal volume of 2-propanol. RNA was purified on ReliaPrep spin columns (Promega, Madison, WI), following the manufacturer’s instructions. RNA concentration and purity of all samples were determined using the LVis Plate function on the FLUOstar Omega microplate reader (BMG Labtech, Aylesbury, UK). RNA was diluted to 50 ng·μL^−1^ and reverse transcribed to cDNA using the nanoScript 2 RT kit and oligo(dT) primers (Primerdesign, Southampton, UK). The resultant cDNA was diluted to 2.5 ng·μL^−1^ before reverse transcription-quantitative polymerase chain reaction (RT-qPCR) analysis. Real-time RT-qPCR analyses were performed in triplicate using Primerdesign custom-designed primers (Primerdesign, Southampton, UK) for GALK1, GALT, and GALE genes (Sigma-Aldrich, St Louis, MO) (Table 3) and Precision*PLUS* qPCR Mastermix with low ROX and SYBR green (Primerdesign, Southampton, UK) on a QuantStudio 5 Real-Time PCR System (Thermo Fisher Scientific, Waltham, MA). For all genes, 5 μL of diluted cDNA (2.5 ng·μL^−1^ concentration) was added to each well containing the appropriate primers and Mastermix for a 20 μL total reaction volume. The qPCR reaction was run according to the manufacturer's instructions (Primerdesign, Southampton, UK), including a melt curve (Thermo Fisher Scientific, Waltham, MA). qPCR results were analyzed using Design and Analysis Software v2.6 (Thermo Fisher Scientific, Waltham, MA).

**Table 3.** Primer sequences

	Gene Description	Primer Sequences (5′–3′)
GALK1	Galactokinase 1	Forward: ATCTAACGGGTCAGGTTGGG
		Reverse: GCAGGTCTGAGTTGAATGCG
GALT	Galactose-1-phosphate uridylyltransferase	Forward: TGACCCTCTCAACCCTCTGT
		Reverse: AGGTGCTATCGTACTGGGGA
GALE	UDP-galactose-4-epimerase	Forward: TTCATGTTGAGGGTCACAGGG
		Reverse: CCTCCTCATTCCTCCTGAGC

### Plasma Analyses

Venous blood samples (∼6 mL) were collected into EDTA tubes, stored on ice, and then centrifuged at 4°C and 1,000 *g* for 15 min. Aliquots of plasma were then stored at −70°C and later analyzed for glucose, lactate, nonesterified fatty acids (NEFA), and glycerol using commercially available kits (Glucose kit, Lactate kit, NEFA kit, Glycerol kit; Randox, London, UK) using an automated photometric clinical chemistry analyzer RX Daytona+ (Randox, London, UK). Plasma galactose concentration was determined using a colorimetric assay (Galactose Assay Kit, Sigma Aldrich, St. Louis, MO) and insulin using an enzyme-linked immunosorbent assay (Invitrogen, Life Technologies, California).

### Statistics

The sample size was estimated from the values taken from a previous study observing muscle glycogen depletion and repletion in response to postexercise carbohydrate ingestion, where basal postglycogen concentration in control (GLU) was 128 (SD 25) mmol·kg^−1^·DM^−1^, and test-retest within-participant error of 9 mmol·kg^−1^·DM^−1^·h^−1^ (9). Using standard equations for crossover trials, controlling for type I error (α = 0.05) and type II error (β = 0.8) and applying a moderate standardized difference of 0.5 times the basal SD as value for the smallest important change, yielded a sample size of 9. This sample size which provided power to detect the smallest critical value of change in muscle glycogen concentration of 9.4 mmol·kg^−1^·DM^−1^·h^−1^. Assuming galactose alone would exhibit a similar effect on muscle glycogen as fructose [i.e., muscle glycogen synthesis difference of 21 mmol·kg^−1^·DM^−1^·h^−1^ for a blend of fructose-glucose versus glucose (9)], the sample size was considered sufficient to estimate expected differences in the skeletal-muscle glycogen synthesis rate with combined galactose-glucose compared with glucose or galactose alone.

The effects of treatment on outcomes were estimated from linear mixed models (Proc Mixed, SAS 9.4, Cary, NC). Fixed effects were treatment, period, and sex (dropped for Western blot owing to nonconvergence), with treatment adjusted for baseline (i.e., immediate postexercise sample) concentration/expression for the primary model, and additionally for the glycogen analysis the amount of work in kJ during the glycogen reducing exercise session. Participant was the random effect and any additional error arising from between-sex differences was inestimable and therefore ignored. Glycogen and plasma data were analyzed as the raw difference scores. Western blot data was the raw blot phosphoprotein density/whole protein concentration, each adjusted to the loading control. Blot data were 100 times natural log-transformed to manage heteroscedasticity, with back-log transformation providing outcomes as percent. Data are presented in graphical form as raw means (SD). Statistical summaries are presented in tabular form expressed as baseline adjusted least squares mean estimates and 95% confidence limits (CLs).

## RESULTS

### Glycogen-Reducing Exercise Sessions

Participants completed 1 226 ± 716, 11 34 ± 458, and 1 148 ± 616 kJ of mechanical work during the glycogen reducing sessions in GLU, GAL, and GAL + GLU, respectively, without any statistically significant differences between the trials (*P* = 0.941). Neither were there any differences in the number of completed stages at 90%, 80%, and 70% Wmax between all three conditions (*P* = 0.769).

### Muscle Glycogen

Means (SD) muscle glycogen concentration measured in rested participants during the familiarization session was 472 (123) mmol·kg DM^−1^, which is in consistent with data from a recent meta-analysis for this population (25).

**Figure 1.**
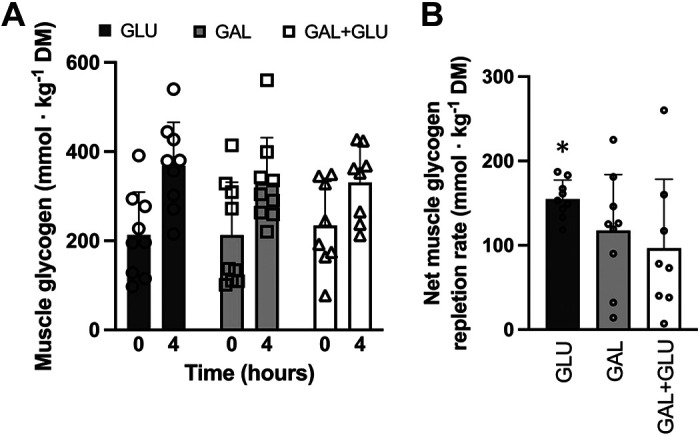
Raw skeletal-muscle glycogen concentration (*A*) immediately (time = 0) and 4-h postexercise and (*B*) net change in skeletal-muscle glycogen concentration over the 4-h recovery period. Data are raw means (SD). All data are *n* = 9 except for the 4-h postexercise sample in GAL + GLU (*n* = 8), where a participant withdrew part way through the recovery associated with gastrointestinal discomfort. *Significantly different between GLU and the other two conditions (GAL and GAL + GLU).

Figure 1 and Table 4 display the statistical estimates for absolute and 4-0-h net changes in skeletal-muscle glycogen content during recovery from exercise.

**Table 4.** Statistical summary of net change in skeletal-muscle glycogen concentration during the 4-h postexercise recovery period

	Mean 4-0 h Estimate (95% CL)			Treatment Effect (95% CL)		
Trial, Contrast	GLU	GLU + GAL	GAL	GAL + GLU – GLU	GAL – GLU	GAL + GLU – GAL
Glycogen concentration, mmol·kg DM^−1^	163 (133, 193)	114 (82, 145)	117 (87, 146)	−50 (−89, −10)	−46 (−84, −8)	−3 (−44, 37)
*P* value				0.021	0.024	0.843

The increase in skeletal-muscle glycogen concentration in response to carbohydrate feeding during recovery from exercise was 1.3- to 1.6-fold higher with GLU relative to GAL + GLU and to GAL. There was no significant difference in the net increase in skeletal muscle glycogen between GAL + GLU and GAL.

### Plasma Metabolites during Recovery

**Figure 2.**
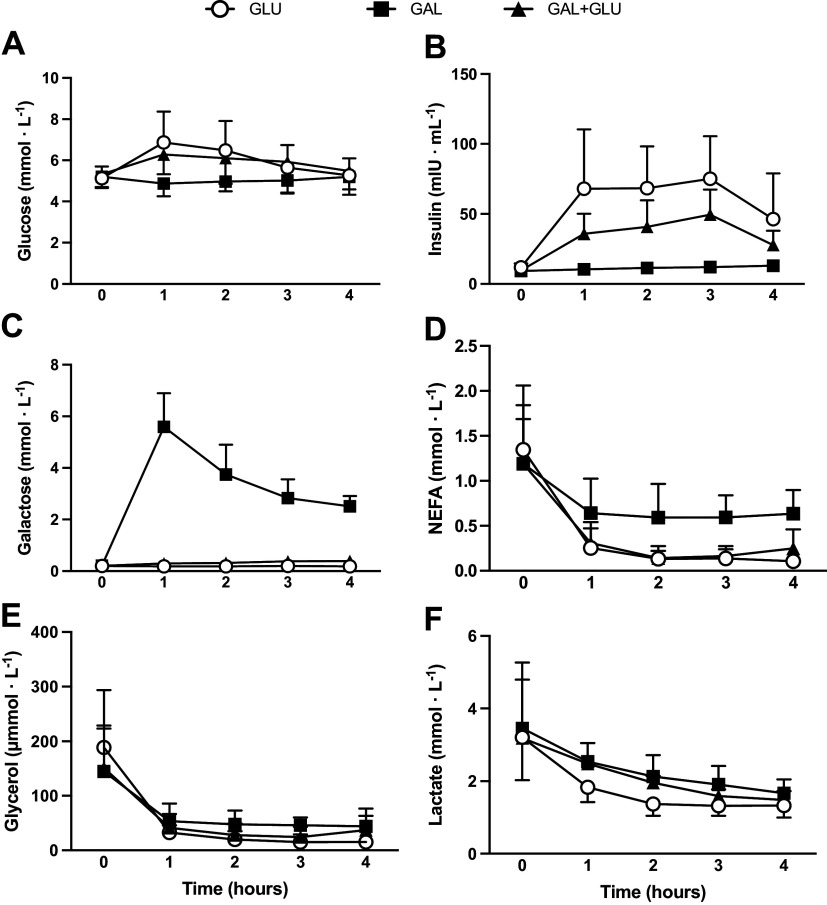
Plasma glucose (*A*), insulin (*B*), galactose (*C*), NEFA (*D*), glycerol (*E*), and lactate concentration (*F*) responses during the 4-h postexercise recovery period. Data are raw means (SD). *n* = 8

The effect of carbohydrate type on plasma metabolite concentrations during the recovery period from exercise is presented in Fig. 2 and Table 5. Blood was obtained from eight participants due to issues with obtaining blood samples from one participant.

**Table 5.** Statistical summary of plasma concentrations during the 4-h postexercise recovery period

	Mean 4-0 h Estimate (95% CL)			Treatment Effect (95% CL)		
Treatment, contrast	GLU	GAL + GLU	GAL	GAL + GLU – GLU	GAL – GLU	GAL + GLU – GAL
Glucose, mmol·L^−1^	0.57 (0.06, 1.08)	0.98 (0.49, 1.47)	−0.19 (−0.81, 0.43)	0.41 (−0.13, 0.94)	−0.75 (−1.34, −0.17)	1.16 (0.53, 1.80)
*P* value				0.134	0.013	0.001
Insulin, µIU·mL^−1^	47 (30, 63)	34 (23, 45)	−1 (−16, 15)	−13 (−28, 2)	−47(−63, -31)	34 (20, 49)
*P* value				0.096	<0.001	<0.001
Galactose, mmol·L^−1^	0.07 (−0.27, 0.40)	0.20 (−0.14, 0.54)	3.46 (3.10, 3.82)	0.13 (−0.11, 0.37)	3.35 (3.07, 3.63)	−3.22 (−3.54, -2.90)
*P* value				0.29	<0.001	<0.001
Lactate, mmol·L^−1^	−1.69 (−1.86, −1.52)	−1.24 (−1.43, −1.06)	−1.39 (−1.54, −1.24)	0.30 (0.11, 0.49)	0.44 (0.24, 0.65)	−0.15 (−0.35, 0.06)
*P* value				0.003	<0.001	0.167
NEFA, mmol·L^−1^	−1.11 (−1.18, −1.03)	−1.00 (−1.07, −0.92)	−0.61 (−0.70, −0.51)	0.11 (0.01, 0.21)	0.50 (0.40, 0.61)	−0.40 (−0.51, −0.29)
*P* value				0.037	<0.001	<0.001
Glycerol, µmol·L^−1^	−141 (−150, −132)	−121 (−130, −113)	−105 (−115, −94)	20 (9, 31)	37 (25, 48)	−17 (−29, −5)
*P* value				<0.001	<0.001	0.007

The plasma glucose response was similar between GLU and GAL + GLU, but 0.5 to 1.8 mmol·L^−1^ higher compared with GAL (Fig. 2*A*). Plasma insulin concentrations stayed unchanged in GAL, but increased in GLU and GAL + GLU, without being different between each other in GLU and GAL + GLU (Fig. 2*B*). Plasma galactose concentrations increased only in GAL peaking at the 1-h time point but remained low throughout the recovery in GAL + GLU and GLU (Fig. 2*C*). Plasma NEFA and glycerol concentrations were suppressed after the carbohydrate feeding was initiated but were least suppressed in GAL feeding as compared with GLU or GAL + GLU (Fig. 2, *D* and *E*). Plasma lactate concentrations were slightly higher in GAL and GAL + GLU feeding as compared with GLU, but not different between GAL and GAL + GLU (Fig. 2*F*).

### Western Blotting and qPCR

**Figure 3.**
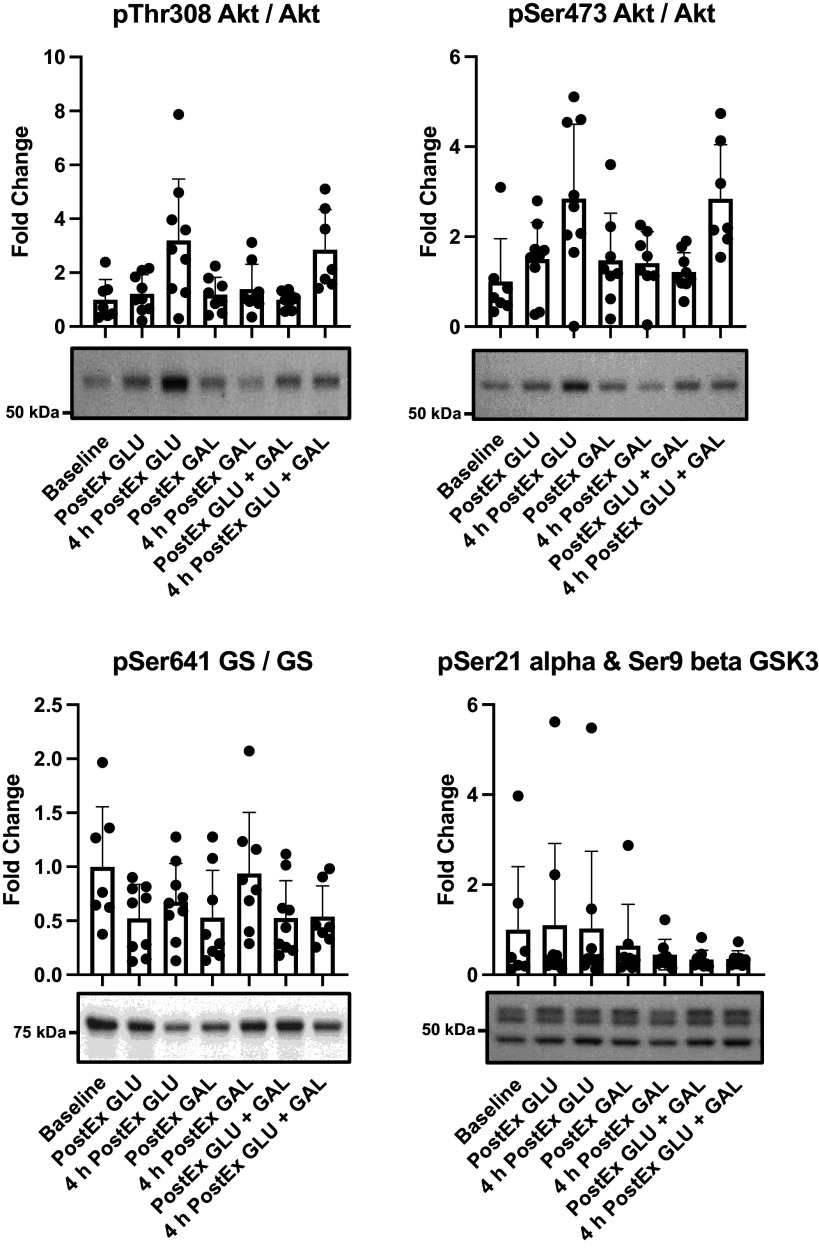
The phosphorylation status of pThr308 Akt/Akt, pSer473 Akt/Akt, pSer641 GS/GS, and pSer21 alpha and Ser9 beta GSK3 with a representative image example. Data are the raw mean phosphoprotein to total protein ratio and SD as error bars. *n* = 8.

Western blotting outcomes are presented in Fig. 3 and Table 6. The change (increase) in phosphorylation of Akt^pThr308^ across the recovery period tended to be higher in GAL + GLU and GLU, relative to GAL without being different between GAL + GLU and GLU. There were no clear differences in the change in phosphorylation of Akt^pSer473^ between the conditions. There appeared to be no clear differences in the increase of glycogen synthase (GS) phosphorylation at pSer641 between any of the conditions, and there were no clear differences in the change in glycogen synthase kinase (GSK) phosphorylation at PSer21 alpha and Ser9 beta.

**Table 6.** Statistical summary of Western blotting outcomes during the 4-h postexercise recovery period

	Treatment Effect (95% CL)		
Trial, contrast	GAL + GLU – GLU	GAL – GLU	GAL + GLU – GAL
pThr308 Akt/Akt	9 (−52, 146)	−53 (−78, 4.3)	129 (−4, 442)
*P* value	0.831	0.062	0.059
pSer473 Akt/Akt	44 (−67, 522)	−31 (−83, 170)	109 (−55, 874)
*P* value	0.601	0.567	0.319
pSer641 GS/GS	−3.4 (−50, 86)	53 (−17, 180)	−37 (−69, 27)
*P* value	0.091	0.156	0.182
PSer21 alpha & Ser9 beta GSK3	−21 (−71, 115)	7 (−66, 238)	−26 (78, 153)
*P* value	0.621	0.904	0.607

Data are presented as % changes from immediate postexercise to 4-h postexercise, with the immediate postexercise value used as a baseline covariate. LSM, least squares means. GLU, *n* = 9, GAL + GLU and GAL, *n* = 7.

**Figure 4.**
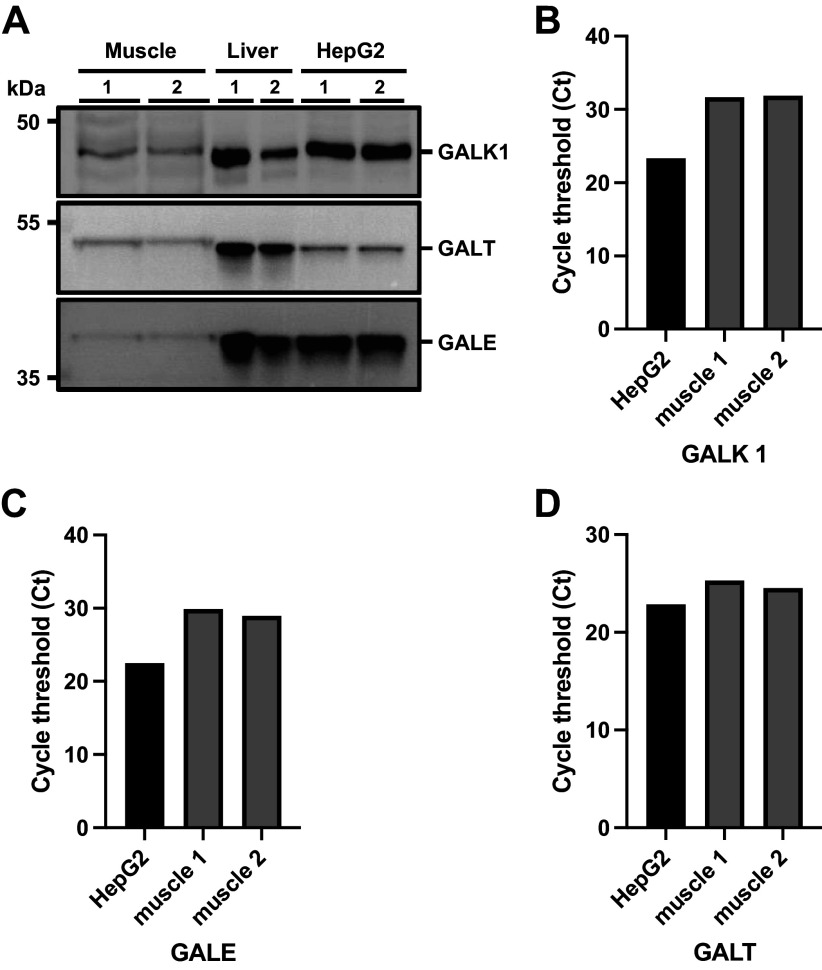
*A*: representative Western blot images of galactokinase 1 (GALK1), UDP-galactose-4-epimerase (GALE), and galactose-1-phosphate uridylyltransferase (GALT) protein expression in human skeletal muscle tissue (60 µg), human liver tissue (10 µg) and HepG2 cells (30 µg). GALK1 antibody specificity was validated in C2C12 myoblasts using the CRISPR-Cas9 knockout method with details and full blots for all Western analysis available as Supplemental material at https://doi.org/10.25500/edata.bham.00000896. Bar graphs showing GALK1 (*B*), GALE (*C*), and GALT (*D*) gene expression as reported by individual cycle threshold (Ct) values in HepG2 cells and human skeletal muscle tissue.

Results of the exploratory qPCR and Western blotting analyses looking into expression of the selected enzymes of the Leloir pathway are presented in Fig. 4. qPCR analysis showed expression of genes GALK1, GALE, and GALT in both HepG2 cells and human skeletal muscle tissue (Fig. 4). The figures from the Western blot show bands at the molecular weight of galactokinase 1 (GALK1), UDP-galactose-4-epimerase (GALE) and galactose-1-phosphate uridylyltransferase (GALT) protein in all samples (Fig. 4).

## DISCUSSION

The principal objective of the present study was to test the hypothesis that glucose and combined galactose-glucose ingestion would elicit similar muscle glycogen resynthesis after a glycogen-reducing exercise bout, and this would be higher than that observed with ingestion of galactose alone. In contrast with the hypothesis, galactose-glucose ingestion resulted in lower muscle glycogen synthesis as compared with the ingestion of an isocaloric amount of glucose. As hypothesized, exclusive ingestion of galactose resulted in lower muscle glycogen synthesis as compared with glucose. Unexpectedly, galactose-only ingestion resulted in comparable muscle glycogen synthesis to combined galactose-glucose ingestion.

The observation that the skeletal-muscle glycogen synthesis rate was lower with the GAL + GLU monosaccharide combination versus GLU contrasts the initial hypothesis which was based on previous findings showing similar glycogen resynthesis responses with fructose-glucose versus glucose ingestion (9, 10) as both galactose and fructose are thought to require hepatic conversion to glucose and systemic transportation and uptake before contributing to skeletal muscle glycogen formation (18, 21). One of the potential mechanisms for lower skeletal-muscle glycogen resynthesis in GAL + GLU as compared with GLU could be intestinal transport limitation as both galactose and glucose use the same intestinal transporters, whereas fructose and glucose are transported by different transporters. However, this would not explain why fructose-glucose and glucose only at high ingestion rates result in similar postexercise muscle glycogen synthesis (9, 11), despite fructose affording the additional intestinal transport capacity resulting in higher exogenous carbohydrate oxidation rates during exercise (26). Furthermore, postexercise liver glycogen replenishment was similar using fructose-glucose and galactose-glucose combination (13), again despite activating multiple versus single intestinal transport pathways. Therefore, it seems unlikely that intestinal transport limitation explains why fructose-glucose, but not galactose-glucose, elicits similar postexercise muscle glycogen synthesis to glucose only.

While there were no significant differences in plasma glucose responses to GAL + GLU or GLU, plasma insulin concentration was on average 27% lower in GAL + GLU (Fig. 2*B*, Table 5). Lower insulin relative to GLU is consistent with the results of a previous study that investigated postexercise liver glycogen synthesis in response to ingestion of glucose and maltodextrin, fructose and maltodextrin, and galactose and maltodextrin mixtures (13). Differences in insulin have previously been shown to contribute to variations in skeletal-muscle glycogen synthesis rates (27, 28). The reduced muscle glycogen synthesis was observed with GAL + GLU versus GLU in the present study could, therefore, be explained in part by a lower insulin response, although more data or sample size would be required to reduce statistical uncertainty. It should be noted, however, that no clear differences were observed between GAL + GLU and GLU in a downstream measure of activation of the skeletal-muscle insulin-signaling cascade, namely phosphorylation of Akt^Thr308^ and Akt^Ser473^ (Table 6) (29). Furthermore, lower skeletal-muscle glycogen synthesis rate was observed in GAL-GLU versus GLU despite no clear differences between conditions in a marker of skeletal muscle glycogen synthase activity (i.e., phosphorylation of GS^Ser641^; Table 6) (30). Although it cannot be discounted that insulin could still have played a role irrespective of the enzyme phosphorylation sites, based on the present data it is not possible to attribute a clear mechanism(s) explaining the inferior response of muscle glycogen to GAL-GLU versus GLU.

Muscle glycogen synthesis was lower with GAL compared with GLU, which was expected based on the presumed predominance of hepatic galactose metabolism and the typically observed modest glycemia and insulinemia observed after galactose ingestion (21, 31). Indeed, changes in glucose and insulin concentrations have previously been shown to explain 94% of variance in muscle glycogen replenishment (32). Of note is that glycogen synthesis responses in GAL + GLU and GAL appear more variable than those seen in GLU. Nonetheless, on average GAL did elicit changes in muscle glycogen concentration to a magnitude like that observed in the GAL + GLU condition. This observation is particularly interesting when one considers that 0.8 g·kg^−1^·h^−1^ of glucose (i.e., direct substrate for muscle glycogen synthesis) was provided in GAL + GLU. Furthermore, there were marked increases in plasma glucose and insulin in GAL + GLU yet no discernible changes were observed in GAL. Therefore, the data reveal an appreciable effect of galactose ingestion in the apparent absence of traditionally considered key regulators of postexercise muscle glycogen synthesis (i.e., ingested glucose, glycemia, and insulinemia).

A key outstanding issue arising from these data relates to the mechanism(s) by which postexercise muscle glycogen synthesis was appreciably facilitated in GAL. The absence of discernable changes in plasma glucose during the recovery period argues against a substantial contribution from the indirect pathway (i.e., hepatic galactose to glucose conversion via the Leloir pathway with subsequent direct muscle glucose uptake and glycogen synthesis). Furthermore, the lack of change in plasma insulin concentration or muscle Akt phosphorylation (Thr308 and Ser473) suggests that insulin-stimulated glucose uptake to facilitate glycogen synthesis was not a dominant factor. One consideration is the potential for direct skeletal muscle galactose uptake and subsequent conversion to glycogen. Indeed, galactose could be transported via GLUT4 (33), the presence of which is increased at the muscle membrane in the postexercise period independently of insulin (34). Earlier studies conducted on eviscerated dogs and rats, respectively, provided evidence for peripheral (assumed muscle) galactose uptake in a similar mechanism as glucose (i.e., with insulin stimulation, which would invoke GLUT4-induced galactose/glucose uptake) (35, 36). In the present study, plasma galactose concentrations on average peaked at 5.6 mmol·L^−1^ and remained substantially elevated throughout the recovery period (on average to ∼3.5 mmol·L^−1^), whereas this was not the case in GAL + GLU condition, likely due to enhanced splanchnic galactose extraction due to glucose coingestion (37). Thus, a high concentration gradient was created, which is important as glucose transport through GLUT4 (34), and by inference galactose uptake through GLUT4, also operates in proportion to diffusion-gradient mediated flux kinetics.

After uptake into skeletal muscle cells, galactose would need to be phosphorylated to glucose-6-phosphate to be able to be converted to glycogen. Skeletal muscle is not typically considered as a site for galactose utilization. However, the activity of two enzymes of the Leloir pathway (GALK1 and GALT) has been detected in human skeletal muscle (38, 39). Human muscle primary cells can be differentiated in galactose medium, suggesting galactose use as an energy source (40). Our exploratory analyses demonstrate the gene and protein expression of GALK1 and GALT in human skeletal muscle, but also GALE, another galactose enzyme in the Leloir pathway. Interestingly, similar observations apply to fructose in that it can be taken up, oxidized, and stored as glycogen in human skeletal muscle under condition of elevated blood fructose concentration (15–17). Our observations suggest further work should investigate the capacity for and significance of galactose metabolism in human skeletal muscle.

In conclusion, this study demonstrated that postexercise galactose-glucose coingestion results in a lower rate of skeletal-muscle glycogen replenishment compared with exclusive glucose-only ingestion. The observation of noteworthy postexercise muscle glycogen synthesis with exclusive galactose-only ingestion without discernible elevations in plasma glucose and insulin concentrations, and evidence for skeletal-muscle expression of Leloir pathway enzymes, suggests galactose could be a direct metabolic substrate for glycogen restoration postexercise and warrants further investigation.

## DATA AVAILABILITY

Data will be made available upon reasonable request.

## SUPPLEMENTAL DATA

10.25500/edata.bham.00000896 Data supplements can be accessed at: https://doi.org/10.25500/edata.bham.00000896.

## GRANTS

This project was supported by a grant from Dairy Management Inc. to G.A.W. and D.S.R., which comprises the National Dairy Council, The American Dairy Association, and the U.S. Dairy Export Council. TP was supported by a scholarship from the Public Scholarship, Development, Disability, and Maintenance Fund of the Republic of Slovenia.

## DISCLAIMERS

This study includes independent research supported by the Birmingham National Institute for Health Research (NIHR) Birmingham Biomedical Research Center, based at the University of Birmingham. The views expressed are those of the authors and not necessarily those of the NHS, the National Institute of Health Research or the Department of Health and Social Care.

## DISCLOSURES

T.P. has acted as a consultant for a sports nutrition brand Nduranz (Slovenia). O.J.O. has received funding from Volac International (United Kingdom) as part of an iCASE PhD studentship in partnership with the Biotechnology and Biological Sciences Research Council (United Kingdom). G.A.W. has received research funding and/or has acted as a consultant for GlaxoSmithKline Ltd (United Kingdom), Sugar Nutrition UK, Lucozade Ribena Suntory Ltd (United Kingdom), and Volac International Ltd. D.S.R. has received consultancy research funds from Frucor Suntory Beverages (New Zealand), Zespri Ltd (New Zealand), Lucozade-Ribena-Suntory Ltd (United Kingdom). None of the other authors has any conflicts of interest, financial or otherwise, to disclose.

## AUTHOR CONTRIBUTIONS

Y.-C.L., D.S.R., and G.A.W. conceived and designed research; T.P., B.J.S., A.P.S., O.J.O., S.O.L., R.C., R.B.S., E.L.S., P.F.L., Y.S.E., and G.A.W. performed experiments; T.P., B.J.S., A.P.S., O.J.O., R.C., D.S.R., and G.A.W. analyzed data; T.P., A.P.S., R.C., Y.-C.L., D.S.R., and G.A.W. interpreted results of experiments; T.P., A.P.S., and R.C. prepared figures; T.P. and G.A.W. drafted manuscript; T.P., B.J.S., A.P.S., O.J.O., P.F.L., D.S.R., and G.A.W. edited and revised manuscript; T.P., B.J.S., A.P.S., O.J.O., S.O.L., R.C., R.B.S., E.L.S., P.F.L., Y.S.E., Y.-C.L., D.S.R., and G.A.W. approved final version of manuscript.
